# Effects of Non-Thermal Plasma Treatment on Plant Physiological and Biochemical Processes

**DOI:** 10.3390/plants11081018

**Published:** 2022-04-08

**Authors:** Vida Mildaziene, Bozena Sera

**Affiliations:** 1 Faculty of Natural Sciences, Vytautas Magnus University, LT-44404 Kaunas, Lithuania; 2 Department of Environmental Ecology and Landscape Management, Faculty of Natural Sciences, Comenius University in Bratislava, 84215 Bratislava, Slovakia; bozena.sera@uniba.sk

Plasma, also called the fourth state of matter, is partially or fully ionized gas. A distinction is made between thermal (high temperature, equilibrium) and non-thermal (cold, low temperature, non-equilibrium) plasma. Thermal plasma reaches temperatures of thousands of kelvin and occurs in the Sun, lighting, electric sparks, tokamaks, etc. Non-thermal plasma (NTP) occurs at near-ambient temperatures, and its high kinetic energy is only stored in electrons [1,2].

NTP can be implemented at low (using expensive vacuum equipment) or atmospheric pressure (a simpler and cheaper option). NTP at atmospheric pressure is of considerable practical interest because it allows one to avoid the use of expensive vacuum equipment and can be used for the treatment of exhaust gases and polluted liquids. The latter cannot be realized at low pressure. Another advantage of atmospheric-pressure NTP, over low-pressure NTP, is its ability to intensify plasma treatment and induce rapid changes in treated materials [3,4].

NTP at atmospheric pressure has wide applications in biology, medicine, dermatology, dentistry, agriculture, forestry, and the food industry [5,6,7,8,9,10,11]. The possible applications include disinfection processes, the acceleration of blood coagulation, the improvement of wound healing and infection clearance, dental applications, and cancer therapy [12]. Applications of both low-pressure and atmospheric-pressure NTP based on various effects observed in plants have also been widely reported, and the number of publications has been increasing in recent years. The topics include changing seed-surface properties, microbial seed decontamination, the degradation of mycotoxins, the stimulation of seed germination and seedling growth, inducing changes in metabolic plant pathways, modulating the enzymatic activities of enzymes, modulating the contents of phytohormones, inducing stress resistance, and influencing productivity [13,14,15,16].

The application of NTP in agriculture includes both direct and indirect means of NTP treatment. A quantity of published data demonstrate mechanisms of direct interactions between plasma and plant material [17]. Indirect NTP treatments are used less often, it is usually the application of plasma-activated water (PAW) to the plant [18,19].

The potential of NTP applications in sustainable agriculture is supported by numerous studies. The experimental evidence gathered to date suggests that the plasma treatment of seeds, water or plants can be used to improve yields, increase the size and robustness of plants, and reduce the need for antifungal agents, as well as other chemicals. However, the development of reliable and manageable agro-biotechnologies is ultimately based on the understanding of the molecular mechanisms underlying such effects. Despite considerable efforts, such knowledge still remains elusive.

This Special Issue of the *Plants* journal aims to present the most recent findings on changes in plant signal transduction, metabolism, development, and physiological processes induced by the exposure of seeds or plants to cold plasma or PAW and leading to increased plant productivity. This Special Issue includes a number of original studies on the effects of NTP on plants that can be divided into three groups: (1) articles presenting experimental studies on the direct action of NTP on seeds or plants; (2) results obtained using PAW; (3) literature reviews.

Direct action of NTP (1). The potential of pre-sowing seed treatment with low-pressure capacitively coupled NTP and radiofrequency electromagnetic fields (EMFs) for the stimulation of the biosynthesis of steviol glycosides, stevioside (Stev) and rebaudioside A (RebA) in stevia plants was studied by Judickaite et al. [20]. In this study, the impact of treatments on seed germination and the content of other secondary metabolites—phenolic compounds and flavonoids—as well as the subsequent effects on antioxidant activity were also investigated. It was demonstrated that short pre-sowing treatments of stevia seeds with low-pressure NTP and EMFs could be a powerful tool for the enhancement of the biosynthesis/accumulation of RebA and Stev. However, the applied treatments decreased the RebA/Stev ratio, phenolic and flavonoid contents, and antioxidant activity.

Starič et al. [21] studied the impact of direct (glow) and indirect (afterglow) radiofrequency (RF) oxygen plasma treatments (a type of NTP) on the germination and early growth of two winter wheat varieties (*Triticum aestivum*, var. Apache and var. Bezostaya 1). The germination rate, number of roots, length of the root system, and fresh weight of the seedlings were measured. Both positive and negative differences between the two wheat varieties were observed. Overall, it can be concluded that, besides the NTP treatment parameters, the plant variety and/or seed characteristics may play a crucial role in optimizing NTP for seed treatment, as has previously been demonstrated for other plant species (e.g., [22,23,24]).

The authors of [25] studied the effect of NTP on the fresh and dry biomass production of an ornamental gerbera plant (*Gerbera jamesonii* ‘Babylon F1’) grown in peat or green compost using a standard or a low-fertilization regime. The different types of treatments and their combinations were evaluated to assess possible improvements in plant nutrition, yield, and ornamental traits, as well as variations in the presence of microorganisms in the rhizosphere, when using NTP to treat the nutrient solution. The NTP treatment promoted fresh leaf and flower biomass production in plants grown in peat as well as nutrient adsorption in plants grown in both substrates, except for Fe, while decreasing the dry plant matter. The results revealed that peat, along with an NTP-treated solution containing a high concentration of nutrients, is the most efficient combination of treatments. On the other hand, according to Cannazzaro et al. [25], the combination of NTP and compost may not be suitable for this type of ornamental species.

Using of PAW pre-treatment (2). In an experiment by Cortese et al. [26], the signaling mechanisms behind the effects induced by PAW in *Arabidopsis thaliana* seedlings were investigated. The potential involvement of calcium in the plant’s response and signal transduction induced by the molecules contained in PAW was evaluated. By using an *Arabidopsis* line expressing the bioluminescent Ca^2+^ reporter aequorin in the cytosol, the authors demonstrated that PAW evoked rapid and sustained cytosolic Ca^2+^ elevations, characterized by specific signatures. These signatures depend on several parameters: (a) the operational conditions of the torches used to generate the PAW; (b) the duration of H_2_O exposure to plasma; (c) the dose of PAW administered to the plants; and (d) the temperature and duration for the PAW storage. The authors concluded that the magnitude of the recorded Ca^2+^ signals was dependent on the torch power or the energy transferred to the water during PAW generation. The obtained data suggest that the PAW-induced Ca^2+^ signature may be attributable to a complex “cocktail” of different reactive chemical species contained in PAW, rather than to a single component. This work provides evidence that Ca^2+^ acts as an intracellular messenger in the signaling pathway triggered by PAW in *Arabidopsis*. Understanding the signaling mechanisms behind the plant’s response to PAW is important for its applications in agriculture, with the potential for enabling more sustainable agriculture.

For the first time, Danilejko et al. [27] introduce a manufactured plasma-chemical reactor for the rapid activation of large volumes of liquids (PAW). In their study, the antifungal activity of PAW was investigated. The authors demonstrated that PAW significantly reduced the amount of phytopathogens in sorghum of the Estonskoe variety (*Sorghum bicolor*), wheat (*Triticum aestivum*) seeds, and strawberry (*Fragaria* sp.) fruits. They confirmed a positive effect of PAW on sorghum seed germination and grain biomass production. In addition, a positive effect of PAW on sorghum drought tolerance in a saline semi-desert region was discovered under field conditions.

In the study presented by Lukacova et al. [28], PAW generated by a transient spark discharge operating in ambient air was used on maize (*Zea mays*, hybrid Bielik) corns and seedlings (cultivated for 3 days in paper rolls and 10 days via hydroponics). The roots and shoots were analyzed for guaiacol peroxidase (POX) activity; the root tissues were analyzed for their lignification, and the root cell walls, for in situ POX activity. The activity of POX and catalase after the arsenic-stress treatment of the seedlings was examined, along with the concentration of the photosynthetic pigments in the leaves, and the concentrations in the leaves and roots. The results of this relatively complex experiment suggest that PAW has a positive effect on the physiological responses in maize corns and young plants under arsenic stress.

The work of Kostolani et al. [17] aimed to investigate the effect of PAW on three-day-old pea (*Pisum sativum* cv. Eso) and barley (*Hordeum vulgare* cv. Kangoo) seedlings by estimating the PAW-induced differences in growth and physiological parameters: the germination dynamics; growth parameters; the total soluble protein concentration; the activity of hydrolytic enzymes, antioxidant enzymes, and dehydrogenases; and the visualization of reactive oxygen species and the level of DNA damage. The authors found that PAW generated by glow discharge could have a positive effect on most of the measured parameters. They concluded that the concentrations of stable reactive oxygen–nitrogen species in PAW, favorable for accelerating the transition to aerobic metabolism in the pea, may not be suitable for different plant species, such as barley [17]. The authors suggest that the response to treatment depends not only on PAW but also on the plant species.

Reviews (3). Three reviews included in the Special Issue focus on the effects of seed/plant treatment with NTP [29,30,31]. An overview of the existing knowledge on changes in both biochemical and physiological processes induced by seed treatment with NTP was published by Mildaziene et al. [29]. The effects of seed treatment with NTP on DNA methylation, wide-scale changes in gene and protein expression, and enzyme activities in the affected seeds and growing plants are considered in the context of the observed effects on plant growth and yield. Particular attention is paid to the importance of seed dormancy, the role of reactive oxygen and nitrogen species and phytohormones, the mobilization of secondary metabolism, increased adaptability to stress, and the effects on the plant-associated microbiome. This review also outlines possible future research directions.

Other authors focused their review [30] on a group of legumes (*Fabaceae* family) whose representatives (19 species) have frequently been used in experiments examining the effects of NTP or PAW on plants. The paper summarizes and critically evaluates the current results on seed germination and initial seedling growth, surface microbial decontamination, and the induced changes in seed wettability and metabolic activity.

Starič et al. [31] reviewed the main concepts and underlying principles of NTP treatment techniques as well as the various aspects of NTP’s interaction with seeds. Different plasma-generation methods and setups are described, and the impact of NTP treatments on DNA damage, gene expression, enzymatic activities, morphological and chemical traits, seed germination and plant resistance to stress is considered. Important parameters of the NTP and the interactions of plasma species with the seed surface are presented and discussed.

Conclusions. Recent breakthroughs in research on the effects of NTP seed treatments on plants are strongly linked to discoveries in plant physiology and biochemistry and are related to plant plasticity, adaptability, stress responses and communication. Short NTP treatments of plant materials can induce various changes in plant development and metabolism that persist for a long time. We are only beginning to understand how to use very complex molecular mechanisms for the mobilization of plant resources and for the improvement of agricultural plant performance. It is likely that investigations of plasma-induced changes in plant physiological and biochemical processes may reveal new facts of both fundamental and applied importance.

## Data Availability

All the data included in the main text.

## References

[1] Piel A. (2010). Plasma Physics. An Introduction to Laboratory, Space, and Fusion Plasmas.

[2] Shintani H., Sakudo A. (2016). Gas Plasma Sterilization in Microbiology: Theory, Applications, Pitfalls and New Perspectives.

[3] Tendero C., Tixier C., Tristant P., Desmaison J., Leprince P. (2006). Atmospheric Pressure Plasmas: A Review. Spectroc. Acta B.

[4] Akishev Y., Grushin M., Karalnik V., Kochetov I., Napartovich A., Trushkin N. (2010). Genaration of atmospheric pressure non-thermal plasma by diffusive and constricted discharges in air and nitrogen at the rest and flow. J. Phys. Conf. Ser..

[5] Fridman G., Brelles M.G. (2012). Non-equilibrium Plasmas in Biology and Medicine. Biological and Environmental Application of Gas Discharge Plasmas.

[6] Graves D.B. (2012). The Emerging Role of Reactive Oxygen and Nitrogen Species in Redox Biology and Some Implications for Plasma Applications to Medicine and Biology. J. Phys. D Appl. Phys..

[7] Attri P., Ishikawa K., Okumura T., Koga K., Masaharu S.M. (2020). Plasma Agriculture from Laboratory to Farm: A Review. Processes.

[8] Pankaj S.K., Wan Z., Keener K.M. (2018). Effects of Cold Plasma on Food Quality: A Review. Foods.

[9] Cheruthazhekatt S., Cernak M., Slavicek P., Havel J. (2021). Gas Plasmas and Plasma Modified Materials in Medicine. J. Appl. Biomed..

[10] Domonkos M., Tichá P., Trejbal J., Demo P. (2021). Applications of Cold Atmospheric Pressure Plasma Technology in Medicine, Agriculture and Food Industry. Appl. Sci..

[11] Paužaitė G., Malakauskienė A., Naučienė Z., Žūkienė R., Filatova I., Lyushkevich V., Azarko I., Mildažienė V. (2018). Changes in Norway Spruce Germination and Growth Induced by Pre-sowing Seed Treatment with Cold Plasma and Electromagnetic Field: Short-Term versus Long-Term Effects. Plasma Process. Polym..

[12] Metelmann H.R., von Woedtke T., Weltmann K.D. (2018). Comprehensive Clinical Plasma Medicine. Cold Physical Plasma for Medical Application.

[13] Ten Bosch L., Pfohl K., Avramidis G., Wieneke S., Wolfgang V.W., Karlovsky P. (2017). Plasma-Based Degradation of Mycotoxins Produced by *Fusarium*, *Aspergillus* and *Alternaria* Species. Toxins.

[14] Scholtz V., Šerá B., Khun J., Šerý M., Julák J. (2019). Effects of Nonthermal Plasma on Wheat Grains and Products. J. Food Qual..

[15] Scholtz V., Jirešová J., Šerá B., Julák J. (2021). A Review of Microbicidal Decontamination and Disinfection of Cereals by Non-thermal Plasma. Food.

[16] Šerá B., Vanková R., Roháček K., Šerý M. (2021). Gliding Arc Plasma Treatment of Maize (*Zea mays* L.) Grains Promotes Seed Germination and Early Growth, Affecting Hormone Pools, but Not Significantly Photosynthetic Parameters. Agronomy.

[17] Kostoláni D., Ndiffo Yemeli G.B., Švubová R., Kyzek S., Machala Z. (2021). Physiological Responses of Young Pea and Barley Seedlings to Plasma-Activated Water. Plants.

[18] Kučerová K., Henselová M., Slováková Ľ., Hensel K. (2019). Effects of Plasma Activated Water on Wheat: Germination, Growth Parameters, Photosynthetic Pigments, Soluble Protein Content, and Antioxidant Enzymes Activity. Plasma Process. Polym..

[19] Ndiffo Yemeli G.B., Švubová R., Kostolani D., Kyzek S., Machala Z. (2021). The Effect of Water Activated by Nonthermal Air Plasma on the Growth of Farm Plants: Case of Maize and Barley. Plasma Process. Polym..

[20] Judickaitė A., Lyushkevich V., Filatova I., Mildažienė V., Žūkienė R. (2022). The Potential of Cold Plasma and Electromagnetic Field as Stimulators of Natural Sweeteners Biosynthesis in *Stevia rebaudiana* Bertoni. Plants.

[21] Starič P., Grobelnik Mlakar S., Junkar I. (2021). Response of Two Different Wheat Varieties to Glow and Afterglow Oxygen Plasma. Plants.

[22] Sera B., Sery M., Gavril B., Gajdova I. (2017). Seed Germination and Early Growth Responses to Seed Pre-Treatment by Non-Thermal Plasma in Hemp Cultivars (*Cannabis sativa* L.). Plasma Chem. Plasma Process..

[23] Mildaziene V., Pauzaite G., Nauciene Z., Zukiene R., Malakauskiene A., Norkeviciene E., Stukonis V., Slepetiene A., Olsauskaite V., Padarauskas A. (2020). Effect of Seed Treatment with Cold Plasma and Electromagnetic Field on Red Clover Germination, Growth and Content of Major Isoflavones. J. Phys. D Appl. Phys..

[24] Ivankov A., Naučienė Z., Degutytė-Fomins L., Žūkienė R., Januškaitienė I., Malakauskienė A., Jakstas V., Ivanauskas L., Romanovskaja D., Slepetiene A. (2021). Changes in Agricultural Performance of Common Buckwheat Induced by Seed Treatment with Cold Plasma and Electromagnetic Field. Appl. Sci..

[25] Cannazzaro S., Traversari S., Cacini S., Di Lonardo S., Pane C., Burchi G., Massa D. (2021). Non-Thermal Plasma Treatment Influences Shoot Biomass, Flower Production and Nutrition of Gerbera Plants Depending on Substrate Composition and Fertigation Level. Plants.

[26] Cortese E., Settimi A.G., Pettenuzzo S., Cappellin L., Galenda A., Famengo A., Dabalà M., Antoni V., Navazio L. (2021). Plasma-Activated Water Triggers Rapid and Sustained Cytosolic Ca^2+^ Elevations in *Arabidopsis thaliana*. Plants.

[27] Danilejko Y.K., Belov S.V., Egorov A.B., Lukanin V.I., Sidorov V.A., Apasheva L.M., Dushkov V.Y., Budnik M.I., Belyakov A.M., Kulik K.N. (2021). Increase of Productivity and Neutralization of Pathological Processes in Plants of Grain and Fruit Crops with the Help of Aqueous Solutions Activated by Plasma of High-Frequency Glow Discharge. Plants.

[28] Lukacova Z., Svubova R., Selvekova P., Hensel K. (2021). The Effect of Plasma Activated Water on Maize (*Zea mays* L.) under Arsenic Stress. Plants.

[29] Mildaziene V., Ivankov A., Sera B., Baniulis D. (2022). Biochemical and Physiological Plant Processes Affected by Seed Treatment with Non-Thermal Plasma. Plants.

[30] Šerá B., Scholtz V., Jirešová J., Khun J., Julák J., Šerý M. (2021). Effects of Non-Thermal Plasma Treatment on Seed Germination and Early Growth of Leguminous Plants—A Review. Plants.

[31] Starič P., Vogel-Mikuš K., Mozetič M., Junkar I. (2020). Effects of Nonthermal Plasma on Morphology, Genetics and Physiology of Seeds: A Review. Plants.

